# Families’ experiences of the Low Arousal Approach: a qualitative study

**DOI:** 10.3389/fpsyg.2024.1328825

**Published:** 2024-03-21

**Authors:** Andrew Austin McDonnell, Andrea Page, Stephanie Bews-Pugh, Karolina Anna Morgalla, Tarendeep Kaur-Johal, Mary Maher

**Affiliations:** ^1^School of Nursing and Midwifery, Birmingham City University, Birmingham, United Kingdom; ^2^Studio 3 Clinical Services Limited, Alcester, United Kingdom

**Keywords:** challenging behavior, autism, Low Arousal Approaches, behavior management training, parent training, parent stress

## Abstract

**Background:**

Parents and carers supporting a family member presenting with behaviors of concern experience heightened stress. The Low Arousal Approach is a crisis management strategy which recognizes that stress, or physiological arousal, can be expressed through behaviors of concern. This approach aims to equip parents and carers to manage behaviors in a person-centered and non-confrontational way. There is a paucity of published research exploring the experiences of families applying this approach.

**Methods:**

Seventeen parents who had received training in the Low Arousal Approach were interviewed to gain their perspectives on supporting their family members using this approach.

**Results:**

Thematic analysis revealed themes relating to parental stress, which was related to external pressures, isolation, family stress, and challenges in their caring role. They described encountering negative narratives relating to self-criticism and negative judgments from others. Training in the Low Arousal Approach was related to being empowered through access to evidence, increased confidence, and increased ability to advocate for their family member’s needs. Low Arousal was described as a *“lifestyle”* that enabled increased coping for the family unit as a whole.

**Discussion/conclusion:**

Findings indicate that it is vitally important to recognize the views of parents and carers, and these are equally as important as the views of professionals. We must understand parents’ and carers’ needs in order to provide adequate support.

## Introduction

Challenging behaviors are a common concern among children and adults with intellectual and developmental disabilities (Bowring et al., 2019; van den Akker et al., 2021; Reyes-Martín et al., 2022; Adams et al., 2023). Challenging behavior or behavior that challenges are blanket terms for several behavioral concerns such as aggression, self-injury and property destruction (Adams et al., 2023). These behaviors can negatively affect a person’s well-being, quality of life and access to services (Gur, 2018). Challenging behaviors are multifaceted and associated with biological, psychological, social, and environmental factors (Bowring et al., 2019; van den Akker et al., 2021). Throughout this paper, the authors use the term ‘behaviors of concern’ instead of challenging behavior (Chan et al., 2012).

Carers experience heightened stress when providing direct support to individuals presenting with behaviors of concern (Rippon et al., 2020). The stress experiences of parents caring for an autistic child are reported as clinically significant (Kiami and Goodgold, 2017). The stress and coping strategies of parents caring for a family member with behaviors of concern are well documented (Hastings and Brown, 2002; Hayes and Watson, 2013; Lai and Oei, 2014; Biswas et al., 2015). One qualitative study of twenty parents’ day-to-day experiences of supporting autistic children identified five themes relating to challenges such as children’s behaviors, judgments from others, lack of support, impact on the family, and coping and the importance of appropriate support (Ludlow et al., 2012). Research also shows that a lack of social support is related to increased parental stress, while high support is associated with parental well-being, positive parent–child interactions, and higher scores for autistic children on developmental tests (Boyd, 2002; Kiami and Goodgold, 2017; Staunton et al., 2023).

Within schools and inpatient services, responses to children and young people presenting with behaviors of concern have often involved behavioral or medical interventions (Benson and Brooks, 2008) or the use of physical restraint. A UK-wide survey of families with a child with disabilities carried out by the Challenging Behaviour Foundation (2019) found that 88% of families reported their child had experienced physical restraint, with 35% reporting this as a regular occurrence. Seventy-one per cent of the 204 respondents reported that their child had experienced seclusion – 21% of those said this was on a daily basis – while 87% of parents reported that their child had been injured during a restrictive intervention. The continued use of restrictive practices on autistic children and young people with learning disabilities is deeply upsetting to practitioners and families alike (McMurray, 2020). Furthermore, Kerns et al. (2022) have identified that the use of restrictive practices may lead to the traumatisation of autistic people.

Training carers in behavioral strategies could be considered an integral intervention in the management of behaviors of concern. Training in Positive Behavior Support (PBS) has been identified as being linked to improvements in children’s behaviors (Durand et al., 2013). In a longitudinal case study over a ten-year period, positive outcomes were reported for a child with autism when a PBS approach was utilized (Lucyshyn et al., 2007). There is some evidence for positive outcomes of interventions delivered ‘by proxy’ to parents/carers of children with trauma (Golding, 2019) and anxiety (Lebowitz et al., 2020). The Preschool Autism Communication Trial (PACT) demonstrates long-term symptom reduction in autistic children following a parent-mediated early intervention (Pickles et al., 2016). Parental training has been shown to decrease behaviors of concern in autistic children (Burrell et al., 2020), in addition to reducing parental stress and improving parental confidence (Iadarola et al., 2017). A qualitative study yielded valuable insights into families’ experiences of applying Positive Behavior Supports in practice, including the impact of the framework on behaviors of concern and barriers to implementation (Botterill et al., 2019). In a qualitative study of preschool children who engaged in persisting behaviors of concern, parents questioned their competencies to manage difficult behaviors (Doubet and Ostrosky, 2015). There is a need for more research to look at how best to support families responding to behaviors of concern (McDonnell, 2019). Advice on the day-to-day management of behaviors would appear to be limited. Yet family-centered support is essential to reducing behaviors of concern and parental stress (Argumedes et al., 2018).

The Low Arousal Approach (McDonnell, 2010, 2019) was developed in the 1990s as a reactive behavior management approach to supporting people with behaviors of concern. The approach was originally defined as a collection of behavior management strategies which focus on the avoidance of ‘confrontation’ (McDonnell et al., 1994). The Low Arousal Approach has developed to include strategies that go beyond crisis management (Elvén, 2010; McDonnell, 2019). The approach draws on evidential links between physiological arousal and psychological stress (Wetherell et al., 2006), which are critical in understanding presentations of behaviors of concern (McDonnell et al., 2019). Stress is understood as transactional in terms of (a) the environment, in that stress occurs when stressors outweigh perceived coping resources; and (b) interpersonal, in that stress arises in interaction with others (Lazarus and Folkman, 1984). Formulation considers an individual’s stressors and coping resources. Interventions include teaching emotional regulation and reducing demands. There is a focus on reflective practice, with carers examining their emotional responses and behaviors, aiming to reduce inadvertent triggers to stress/conflict.

Low Arousal training and supports have been adopted by practitioners and families in a variety of settings. The Low Arousal construct has been applied by a variety of different organizations in several European countries. In special schools in Denmark over a two-year period, a significant reduction in staff injuries was reported (Larsen, 2018). Training has also been shown to increase the confidence of staff in adult services supporting autistic individuals (McDonnell et al., 2008; McDonnell, 2010). A single case study applied the Low Arousal Approach to an individual family (Shinnick and McDonnell, 2003; Hewitt et al., 2015). In this study, the families were taught the principles of the Low Arousal Approach and some physical interventions. Applying these approaches to families who are supporting children and adults with behaviors of concern appears to be a significant trend (Woodcock and Page, 2010). There would appear to be benefits to training families in these approaches (Woodcock and Page, 2010); however, to date, there is little evaluative research on the application of these approaches to families.

Many young people are missed by statutory services due to limited capacity to engage in face-to-face interventions and a lack of reasonable adjustments by services (Anderson et al., 2018; Brede et al., 2022). Meanwhile, the burden of care falls on families who are desperate for help. As an alternative to face-to-face support, this study evaluates a systemic intervention delivered to parents/carers of children and young people with behaviors of concern. The intervention consists of training in the Low Arousal Approach and, in some cases, individualized coaching tailored to specific family needs. Anecdotally, families and supporters often design their own training based on the availability of published material (Elvén, 2010; McDonnell, 2010; Woodcock and Page, 2010; McDonnell, 2019). The approach has also been adopted widely by people supporting difficult behavior for individuals who are autistic (McDonnell et al., 2018). Specifically, we interviewed parents about their experiences of training in applying the Low Arousal Approach with their family member who presents with behaviors of concern. By employing qualitative research methodology, we aimed to examine the unique experiences of families who are experts by their lived experience (van Schalkwyk and Dewinter, 2020; Crane et al., 2021) to improve our understanding of applying this approach in family-based settings.

## Method

### Participants

To be eligible, participants needed to speak English, have received some form of training in the Low Arousal Approach, and have caring responsibility for a family member whom they identified as having ‘behaviours of concern.’ They also needed to be able and willing to discuss personal experiences of caring and reflections on the impact of training.

All participants who took part were parents, henceforth referred to as ‘parents’ rather than participants. A total of 17 parents were interviewed; 13 were interviewed individually, and two interviews took place with 2 parents together. Fifteen identified as female, and two identified as male. All parents were over 18 years old. Eleven resided in the UK, 3 lived in Ireland, and 2 in Canada. All parents interviewed had sole or shared caring responsibility for a son or daughter with behaviors of concern. Low Arousal training was provided by *Studio 3 Training Systems and Psychological Services* or partner organizations. Parents received training inputs through traditional training seminars (online or in person), individualized coaching and clinical support, or a combination of both.

Parents discussed their experiences of applying training in relation to 18 family members, with three families identifying that more than one member had behaviors of concern. Ten family members were identified as adults (over 18 years old), whereas eight were identified as children (under 18 years old). Ten were identified as male and eight as female. The majority of the family members (*n* = 13) had a diagnosis of an Autism Spectrum Condition (ASC). The remaining family members were identified as having an intellectual disability (*n* = 1), sensory processing disorder (*n* = 1) or unspecified (*n* = 3). In an attempt to respect confidentiality, other identified characteristics of the young people and families were kept to a minimum.

### Procedure

Parents/carers known to the researchers through attending a *Studio 3* training course were contacted by email and invited to participate in research in which they would be interviewed about their experiences of training in and applying the Low Arousal Approach. If this was responded to with an expression of interest, they were emailed the ‘Participant Information Sheet’ (Appendix A) and the ‘Research Consent Form’ (Appendix B). The participant information sheet gave information about the purpose of the study, the interview process, potential benefits and risks of participation, confidentiality, audio recording, and the right to withdraw. The consent form summarizes these points.

### Interview process

A semi-structured interview schedule was employed as a guide for researchers when conducting interviews (Appendix C). This enabled researchers to gain information to address the research questions while allowing flexibility to follow up on additional related topics brought by participants (Willig, 2013). The interview schedule contained open-ended questions to explore the perceived value of training received, parents’ application of the Low Arousal Approach, and the perceived impact of the approach on parenting, behaviors of concern, and family dynamics. Interviews varied in duration between approximately 40 min and 1 h 15 min. Consent was gained before recording interviews, which were then transcribed verbatim.

### Data analysis

Analysis was informed by the researchers’ training in psychology (AM, KM, MM, SBP and TJK) and in nursing (AP), and by our perspectives as pioneers, advocates, trainers (AM, AP) and practitioners of the Low Arousal Approach (AM, AP, KM, MM, SBP and TKJ). We analyzed interview transcripts following Braun and Clarke’s (2006, 2019) reflexive thematic analysis. We took a realist stance on data, aiming to ground our analysis in the everyday reality and meanings of participants as they experienced them. In line with this, our process was inductive; we did not try to fit the data into pre-existing themes.

Transcripts were divided between researchers, with at least two researchers initially reading each transcript. Each researcher individually noted anything of interest and emerging ideas. The researchers then came together as a group to read and code all transcripts, and it became apparent that there were many overlaps and similarities between notes. Data was manually organized into codes, the smallest possible meaningful data unit. Data extracts were chosen to represent each code. Finally, we began the process of sorting the coded extracts into themes. This involved thinking about how various codes related to one another to form overarching themes and sub-themes.

### Community involvement

The authors sought advice from experts with lived experience, which led to the choice of a qualitative methodology. One of the authors is a parent with lived experience who is also a Low Arousal practitioner. The study was discussed with a number of parents to identify potential participants.

## Results

**Figure 1:**
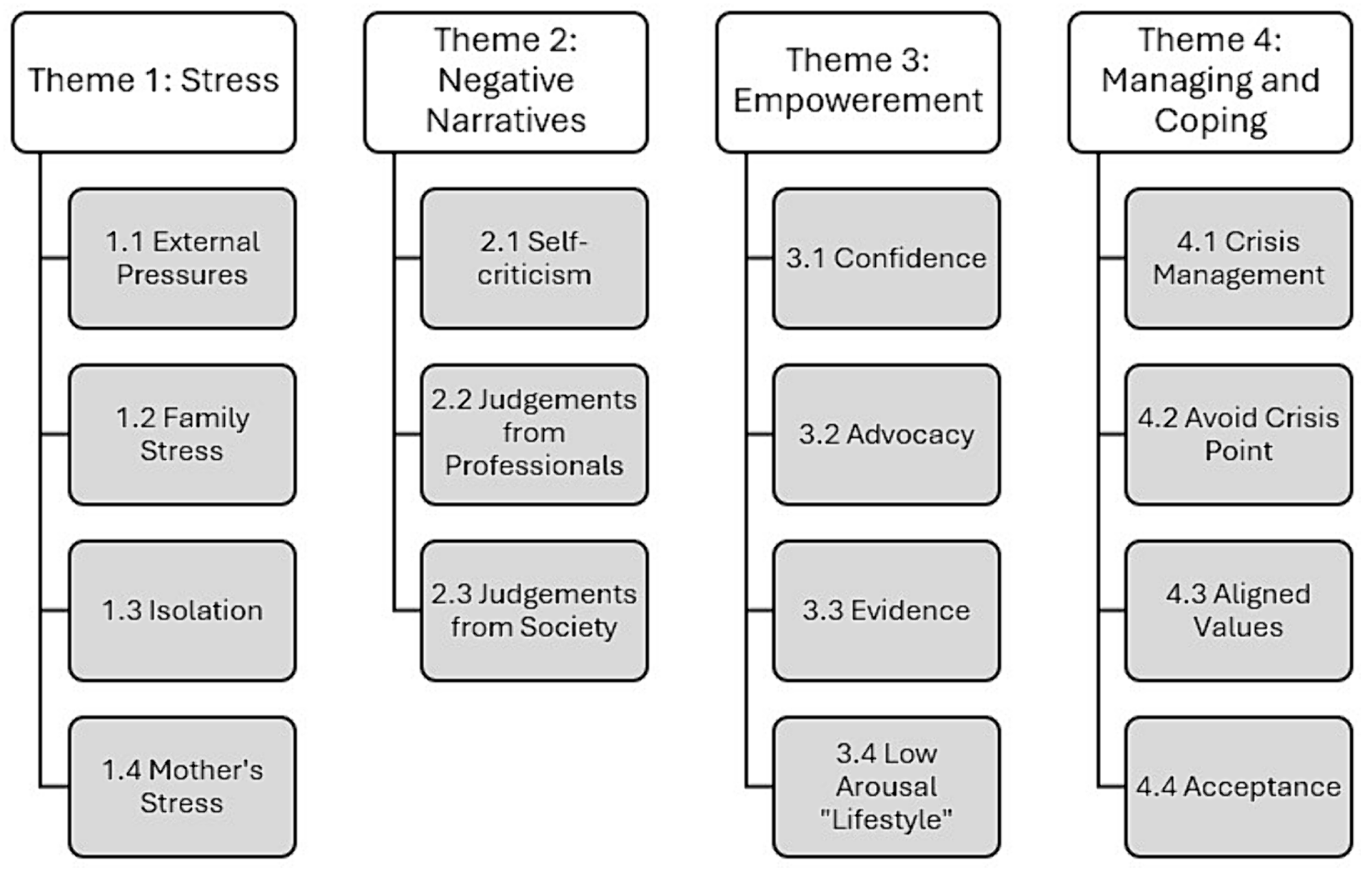
Families experiences of the Low Arousal Approach: themes and sub-themes.

Following analysis, four themes emerged (Figure 1). Parents discussed their experiences of *Stress* (theme 1), *Negative Narratives* (theme 2), *Empowerment* (theme 3) and *Managing and Coping* (theme 4) in relation to supporting a family member who displays behaviors of concern and applying the Low Arousal Approach.

### Theme 1: stress

Many parents (*n* = 13) discussed their high stress levels related to supporting a family member who displays behaviors of concern. These parents suggested that it was a balancing act of meeting external expectations, family needs, and their own needs. Stress appeared to be triggered by external pressures, family stress, and a sense of isolation. Our last sub-theme, ‘Mother’s Stress’, focuses on the role of mothers as the main caregivers and the impact of this role on their stress levels.

*External pressures* (sub-theme 1.1) contributed to the family’s high stress levels. Parents cited their experiences with the *“wider system”* (pp11 q10), such as schools, carers, and local authorities, and the pressures this created for them and their children. One parent said, *“Because you are feeling stressed that you are going to get fined or moaned at, so you apply that stress then onto your child and then your child… cannot cope with that”* (pp1 q14).

Parents have also described that managing external stressors made them *“feel twitchy”* (pp15 q7) and impacted their ability to cope with and manage their family member’s difficulties. The outside pressure made this parent more likely to not *“say the right thing”* (pp15 q7), creating even more escalation.

Furthermore, parents described the challenges of achieving a consistent approach with professionals who do not understand or practise the Low Arousal Approach. One parent felt the need to *“shield the input from others”* to *“offer Low Arousal support in its truest form”* (pp5 q15). While another parent shared that *“applying standard parenting strategies”* is not suitable to their family member’s needs as they *“cannot manage those expectations,”* and it *“leaves a constant friction all the time, and it never goes away”* (pp1 q13).

*Family stress* (sub-theme 1.2) developed from parents describing the impact of their family member’s needs on the family and vice versa. Families with more than one child talked about the struggles of meeting siblings’ needs and the emotional impact on the sibling. Parents described their dilemma as *“who’s needs do you meet?”* (pp6 q9), and often it came down to prioritizing one sibling’s needs *“because it was easier, and it caused less chaos in the family”* (pp6 q9). Another parent reported that *“there was always an element of comparison between the two boys”* (pp9 q14).

Parents also disclosed the exhaustion caused by the frequency and intensity of incidents of ‘challenging’ behavior and associated needs. The family member’s lack of sleep and consequent *“sleep deprivation”* of the parent resulted in overall *“tolerance [going] down”* (pp6 q9). Some parents recalled that without skills to manage these situations, they *“were flying blind all the time”* and *“just needed to physically and emotionally survive the incident”* (pp9 q11). Parental stress impacted their ability to remain objective and empathetic during these incidents. Those highly stressful situations for our participants meant they often lost *“control and understanding of the situation”* (pp8 q10).

*Isolation* (sub-theme 1.3) refers to parental feelings of isolation and lack of effective and compassionate support for themselves and their family member. Families had difficulty asking for help and advocating for their needs as they felt they *“do not have a right to demand anything”* (pp4 q15). One parent disclosed that they were *“falling apart ‘cause [they] did not know where to turn”* or “*how to manage it”* (pp15 q6). Families felt *“really lost as a parent”* (pp9 q16) and *“so isolated”* (pp3 q16). Some had no alternatives to managing incidents at home but to call *“the police”* (pp9 q16). Their sense of being *“helpless to help”* (pp9 q7) their family member created self-blame.

Another sub-theme to emerge from the data was *Mother’s stress* (sub-theme 1.4). Most (*n* = 14) of the participants who took on the main caregiver’s role, and consequently the Low Arousal training, were women. Consistently, the mothers expressed that they were doing most of the care, administration, and crisis management. One mother reported, *“Doing most of the going out with the kids or writing, and if there’s going to be some kind of escalation, it’s usually [them] that faces it”* (pp7 q13). Another mother expressed that this resulted in *“distance”* (pp3 q13) between her and her partner.

Some of our female participants (*n* = 7) felt that their partner’s involvement was *“jarring with [their] approach”* (pp13 q13). This felt *“frustrating”* (pp3, q11). Husbands and partners were described as not “*having the skills to deescalate*” (pp8 q13) due to not having *“done as much research and reading and training and practice”* (pp3, q11), making the training *“difficult to implement”* (pp9 q5).

### Theme 2: negative narratives

This theme emerged as parents described encounters with negative narratives around their parenting and family members. These included instances of self-criticism and judgments from professionals, as well as judgments from family and wider society, related to perceptions of family members’ behaviors and how parents responded. The parents faced negative opinions of their use of the Low Arousal Approach when the approach was misunderstood.

*Self-criticism* (sub-theme 2.1) was noted as parents expressed self-doubt, self-blame, and feelings of helplessness, holding their parenting to account for the family member’s behaviors of concern. One parent expressed how difficult it was to *“separate [family member’s] behaviour from [their] parenting”* (pp9 q7), often blaming self for the incidents of distressed behavior as it *“could have [been] avoided… by doing a, b and c”* (pp7 q 12). For some parents (*n* = 2), the emotional cost associated with challenges around managing incidents triggered personalization and negative self-beliefs about their parenting. Consequently, they felt they were a *“failure”* (pp4 q5) and not *“doing a good job as a parent”* (pp9 q11).

*Judgments from Professionals* (sub-theme 2.2) developed as many families (*n* = 11) felt *“very judged a lot of the time”* (pp3 q16) and that they were given an unhelpful *“label”* (pp15 q16). These negative narratives included blaming parents for their family member’s difficulties. One parent expressed that *“the social workers were coming down on [them] hard, and they obviously blamed parents because… it’s always the parent’s fault… They were quite nasty in it all”* (pp6 q7).

Parents described their experience of professional involvement as lacking empathy when supporting their family members. One reported that they felt *“powerless”* and *“disempowered by [the] school and local authority”* (pp4 q15). Another parent’s family member’s diagnostic status was questioned, and the family was criticized for advocating for recognition of their family member’s neurodiversity. They reported that *“the mental health team saying to [family member] ‘I’m not sure, do you really think autism is your problem? or do you think that your parents are obsessed with it?’”* (pp11 q8).

*Judgments from society and family* (sub-theme 2.3) were noted as families (*n* = 7) felt their parenting methods were judged by extended family, the community, and society as a whole. Parents expressed that they and their children were rarely met with compassion and understanding of their needs. Parents experienced comments about having to be *“harder,” “firmer,”* and *“in control”* (pp6 q8), and that there was a requirement for their family member to *“fit into the normal world”* (pp5 q13). Parents discussed that their family member was perceived as *“self-indulgent”* (pp9 q9), whereas not disciplining their family member *“brought [them] into conflict with people”* (pp1 q9). Consequently, they were asked, *“you sure you should still be parenting?”* (pp9 q16). Another felt they were perceived as a *“wacky parent[s]”* (pp5 q13).

Parents described the impact of perceived societal expectations as affecting their ability to change their parenting methods and meet their family member’s needs. One parent expressed that such expectations are *“very pushed on [us],”* and getting rid of *“all of that conditioning and meet[ing] her where she is”* (pp3 q8) was extremely challenging.

### Theme 3: empowerment

Participants discussed that training in the Low Arousal Approach increased their self-belief and empowered them to refute both self-criticism and negative narratives from others (see Theme 2). Empowerment was related to increased confidence, gaining access to an evidence base, and becoming a stronger advocate for their family member.

Increased *Confidence* (sub-theme 3.1) was noted as parents (*n* = 11) talked about feeling *“much more confident in what we were doing”* (pp9 q11). For some (*n* = 7), this was related to experiencing training as an affirmation of their values and the approach they were already taking with their family member. One parent said that training *“confirmed”* her existing *“beliefs and ideas,”* helping her to *“solidify”* these, giving her *“confirmation that [she] was doing the right thing”* (pp8 q5). As another parent put it, she *“needed that justification… in order to build that confidence”* (pp6 q15). Increased confidence appeared to be related to self-compassion. One parent said, *“You have to have some kind of confidence in order to go, ‘you know what, we have got this!’ And when we have not got this, it’s ok because we will not have it all the time”* (pp6 q15).

*Advocacy* (sub-theme 3.2) emerged as parents talked about increased confidence, enabling them to become a *“stronger advocate”* (pp4 q12) for their family member. The ability to verbalize the Low Arousal Approach meant that parents were able to assert their family members’ needs with professionals, family members, and others. One parent described an incident when her child had been distressed, and she had felt unable to communicate this effectively to the school: *“it’s [not] having words for it…I did not know how to say it”* (pp4 q15). She described that training in the Low Arousal Approach had given her *“the terminology,”* which enabled her to *“feel empowered”* to become a *“stronger advocate for him”* (pp4 q12). Similarly, another parent described that training had equipped her with *“actual word[s]”* (pp7 q11), to be able to inform others, *“this is how we think, this is the practice that we use, here’s the information”* (pp7 q11).

Training appeared to facilitate parents’ recognition of their expertise as the main caregiver. One parent reported, *“I feel like actually, I know what I’m talking about now…. I’m the one who has advocacy for my child in my heart, and I’m the one who knows him best”* (pp4 q15). Another parent discussed that training *“taught [her] to manage the people around [them],”* enabling her to refuse support/interventions that were not suitable to her family member’s needs: *“That’s not suitable, and even if you cannot see that, I’m not putting her through that… I had to learn to stand up for [family member] and tell authority to take a hike”* (pp15 q15).

*Evidence* (sub-theme 3.3) developed as parents (*n* = 9) discussed the Low Arousal Approach having a *“strong theoretic foundation”* (pp11 q8) and *“an actual evidence base”* (pp3 q15). The training gave parents a theoretical framework and body of research they could refer to. They described that this *“quantifiable approach”* (pp13 q5) gave them insight into their child’s emotions and behaviors, and practical methods to manage these. A parent said they valued *“understanding the concept of Low Arousal and why it works and the whole depth of it”* (pp5 q5).

This *“evidence base and the research”* (pp3 q5) was not only utilized to advocate for their family member (sub-theme 3.2), but to advocate for themselves and their approach to parenting. Participants described feeling *“empowered”* (pp4 q15) to engage in discussions with professionals. They described that they had *“good evidence”* (pp5 q13) to *“back up and validate the approach”* (pp3 q5), enabling them to *“stand up”* (pp15 q15) and dispute negative narratives: *“I now have an actual evidence base that I can point people to, to kind of say, well you are entitled to your opinion but please go and do your research”* (pp3 q15). One parent encapsulated this when she said she felt *“more empowered and more knowledgeable” and “knowledge is power”* (pp4 q15).

*Low Arousal lifestyle* (sub-theme 3.4) emerged as parents (*n* = 9) talked about Low Arousal being more than just an approach. Instead, it was described as a *“philosophy”* (pp10 q11), *“embedded in our behaviour”* (pp14 q8), *“our normal strategy”* (pp1 q8), a *“lifestyle”* (pp1 q5 and pp6 q7), or simply *“the way that we live”* (pp1 q7). One parent said, *“It’s more like a style of living rather than we do this as a particular approach. It’s just the way we roll now”* (pp11 q7). This has enabled parents to *“manage life”* (pp6 q7).

Families identified with the values of the approach: *“It’s the kind of philosophy we should all be doing daily in our families”* (pp10 q11). Parents described how the Low Arousal Approach supports family life; one parent said, *“It’s a very kind, very gentle, very supportive way of living your life”* (pp10 q11), while another said, *“It’s just more holistic, natural, family-centred and as I said, we are just five people and a dog trying to live togethe*r*”* (pp2 q16).

### Theme 4: managing and coping

Coping involves adjusting to stressors, and this theme explores how the parents managed caring for their family member using the Low Arousal Approach. Increased coping appeared to be related to managing crisis, reducing stress, having a shared set of values to work to, and acceptance.

Several parents (*n* = 8) discussed applying their knowledge of the Low Arousal Approach when *Managing a crisis* (sub-theme 4.1). Connections were made between exposure to triggers, emotional arousal, and behaviors of concern. One parent described an incident when her son had been exposed to multiple triggers: *“He had broken his arm a couple of days before… he had really bad allergies… [then] we had all the family over… and some of the little younger cousins wanted to watch a different program to what he was watching”* (pp8 q7). She connected this to him becoming *“extremely agitated,”* and to his behavior, which was *“escalating.”* Recognizing that he was *“beyond the point of being able to… compromise”* she described that she applied Low Arousal principles of reducing demands, and of utilizing coping resources to support him to emotionally regulate: *“So, I made sure everybody else left and allowed my son to finished watching the show that he wanted to watch because that was the only thing that was going to help in that situation”* (p8 q5). Another parent discussed her daughter’s physical signs of stress and associated behaviors: *“She immediately turned beet red, the swearing started, some of the flailing,”* which was followed by the parent adjusting her own verbal and non-verbal communication to reduce demands: *“Immediately I… create… more physical distance [and] stop talking”* (pp7 q7).

Parents reflected on the benefit of managing their emotional arousal and how this impacted their family member’s emotions and behaviors: “*Firstly, with yourself, keep calm, and it does have an immediate effect on the whole environment or the situation you are trying to deal with”* (pp17 q5). Parents talked about what their family member’s behaviors may be communicating. One parent discussed *“us[ing] the Low Arousal Approach to really figure out what was going on behind… the meltdown”* (pp3 q7), asking, *“What is his behaviour really communicating and what is the purpose of it? And remembering that you cannot teach a person to swim when they are drowning”* (pp3 11). There appeared to be an increased acceptance (see sub-theme 4.4) of behaviors associated with emotional crises: *“If it does not hurt and the damage can be mended, then it’s not the end of the world”* (pp12 q7).

Many parents (*n* = 14) discussed utilizing the Low Arousal Approach to *Avoid crisis point* (sub-theme 4.2) in the first instance. They described proactively working to *“avoid”* or *“reduce…stres*s*”* (pp6 q7). As when *managing crisis* (sub-theme 4.1 above), parents discussed adapting their communication to meet their family member’s needs, making verbal communication *“softer… shorter”* (pp16 q12) and *“just talk[ing] about the one thing [at a time]”* (pp16 q8). Consequently, it was observed that since using the approach, *“We do not really get the crises situations”* (pp6 q7).

Data suggested that training in the Low Arousal Approach had increased parents’ self-awareness. Parents had the insight to note their thoughts and feelings in relation to their family member’s behaviors of concern and demonstrated how they reframed their perceptions to actively avoid conflict. One parent reported, *“There’s a little part of my brain that goes ‘oh for Christ’s sake, just wash up’ … but… knowing Low Arousal, going no it does not matter it will not take me five minutes to do the washing up… to me it’s not worth upsetting her for”* (pp13 q7). They asked, *“What’s my role in this?”* (pp3 q14), reflected on how they had become *“calmer and less reactive”* (pp2 q12), and observed the effect this had on their family member’s stress levels: *“She does not have meltdowns anymore, and it’s not because she’s changed; it’s because we have changed”* (pp10 q9).

Parents (*n* = 11) discussed that training had given them a framework for their parenting, enabling them to *Align values* (sub-theme 4.3) within the family unit and the wider family. One parent reflected that within the family, *“we all want the same aim in life… we are more aligned in our approach”* (pp14 q13). This enabled families to communicate more effectively, there was more *“willingness to listen”* (pp14 q13), and work together to support their family members, leading to *“improved the whole family atmosphere”* (pp 17 q13).

Adopting the Low Arousal Approach supported extended families to work together and build a shared understanding: *“It’s making life easier for everybody and including all of our family, everybody is very on board, like my extended family they all sort of understand that’s what we need to do to help accommodate my son because he might not be able to handle it, it’s been a life changer”* (pp8 q16). Furthermore, it was discussed that having a shared approach with the Low Arousal practitioners supporting them was a positive experience: *“our values felt very much aligned and that has just never happened before”* (pp2 q16).

Most of the parents (*n* = 15) discussed that their relationship with their family members had improved. Much of this had to do with *Acceptance* (sub-theme 4.4), both becoming more *“accepting of [family member’s] little quirky behaviours”* (pp8 q14) and *“accepting who [the family member] is”* (pp10 q12). Acceptance of behaviors enabled parents to become less reactive: *“I accept certain behaviours now….and actually I do not get affronted”* (pp16 q10). This facilitated *“more empathy”* (pp2 q10) to develop between parents and family members.

Parents demonstrated empathy for their family member’s difficulties: *“He’s struggling in this situation, and we need to support him”* (pp5 q14). Parents reflected that their increased understanding of their family member as a person enabled them to be more adaptive to their needs: *“I’m more receptive to those times when he really wants to talk, and when he’s ready to have a really good conversation with me, so I think I’ve just learnt to understand him and how he thinks better”* (pp8 q11).

## Discussion

This study examined parents’ experiences of using the Low Arousal Approach to managing behaviors of concern displayed by a family member. Four key themes emerged (Theme 1: *Stress*, Theme 2: *Negative Narratives*, Theme 3: *Empowerment*, and Theme 4: *Managing and Coping*), which are discussed below.

Various stressors were experienced by parents. Many reported *Stress* related to being mothers supporting their children with additional needs. Recent research shows that women do still most often take on the main caregivers’ role (Kiami and Goodgold, 2017; Rydzewska et al., 2021), while stress can be cumulative in nature (Weiss et al., 2014), and caring for autistic children is associated with lower marital happiness, family cohesion, and family adaptability (Higgins et al., 2005). This is consistent with our findings, in which female participants described being more actively involved in advocating for their family members and managing incidents at home. The lack of support arising out of being a sole carer with a lack of support from fathers or from their partners was presented as a significant stressor, and appeared to be a potential risk factor for further isolation. This is significant in light of previous findings that many formal support services do not meet parental needs, which contributes to feelings of isolation and alienation (Galpin et al., 2017).

The stressors experienced would appear to be compounded by a consistent finding of the *Negative Narratives* from professionals, family members, and society. Conflicting information about how behavior should be managed was consistent across the accounts of the families. Rules and sanctions were debated, as was the idea of ‘controlling’ children’s behaviors. McDonnell (2019) highlighted that a constant theme in dominant narratives of responses to ‘challenging behaviors’ is what can best be described as ‘the battle for control’. From a parental perspective, it is a disappointing reflection on society that parents still have to justify non-confrontational approaches to managing behavior.

This study was not designed to evaluate training outcomes. It is possible to speculate that training in the Low Arousal Approach did appear to have a positive influence on parental confidence in managing behaviors of concern. The mechanisms underlying these changes may not be attributed solely to training content *per se*. It is likely that there are other processes that may have influenced the partaking families. It is important to note that there was a significant theme of “empowerment” for a number of families in terms of working with their family members. Parents described that training affirmed their key values of non-confrontation and gave them the confidence to apply this with their child despite perceived negative judgments of others. Some parents described themselves as more confident in making a case for these approaches and advocating for their child’s needs. The expression ‘Low Arousal lifestyle’ or similar was utilized across various interviews, which suggests that many parents placed strategies, such as ‘demand reduction,’ almost as a working ‘philosophy.’ Practitioners of the approach have similarly suggested that strategies which avoid day-to-day crises can encourage families to re-evaluate behavioral goals and objectives (Woodcock and Page, 2010).

The final theme focused on *Managing and Coping*. An understanding of the transactional nature of stress and its importance in managing the behavior of autistic children was apparent within the parents’ interviews. They expressed that their empathy for their family member and understanding of reasons behind behaviors of concern had increased. They discussed increased recognition of their family members’ early warning signs that in the past were likely to lead to escalated behaviors, and that this enabled them to adjust their own behavior and respond in a calm and regulated manner. Increased empathic understanding of their family members and increased awareness of their stress-related responses enabled families to avoid reaching crisis points.

The Low Arousal Approach can create ‘internal conflicts’ among people who apply the approach. A typical conflict can involve the avoidance of punitive sanctions and consequences by using demand reduction, which can create a sense in some people that these approaches involve ‘giving in’ (McDonnell et al., 1998). However, themes that have emerged in the current study appear to indicate that training in the low arousal approach has given families the tools to maintain equilibrium within their family unit. With a greater understanding of their own stress, their children’s needs, and internal resources, families felt better able to seek out external support that was appropriate to their family’s needs and supportive of their chosen methods for caring and managing behavior. While some families continued to experience stress, they were able to cope better and avoid crisis situations. Parental perceived self-efficacy and increased levels of perceived control over external stressors may have provided parents with a framework for coping with the difficulties associated with supporting a family member with ‘behaviours of concern’ (Auriemma et al., 2022). In turn, this may have contributed to parents being able to provide the necessary coregulation to support their family members during high-stress situations.

In this study, there appears to be some evidence of improved relationships within some of the families, most notably, greater empathy and acceptance for the family member and their needs. In addition, where families were agreeable with the values of the Low Arousal Approach, greater cohesion and adaptability were reported. On the contrary, family members (husbands, partners, and extended family) who did not understand or actively participate in the application of the approach were distanced from the family member displaying behaviors of concern, the primary caregiver, and in some cases, the whole family.

The consistent theme of *Negative Narratives* from professionals as perceived by families in this study would appear to indicate conflicts between these groups, specifically in terms of providing day-to-day supports and advice. It would be a benefit to focus further on why so many parents seem to feel a lack of support from professionals in this study. This contrasts with parents who, after training, focus on specific day-to-day practice issues. Understanding that the views of families and parents are equally as valid as those of academic practitioners would appear to be an issue that needs to be addressed. It is important to note that some of the statements had a strong ‘anti-professional’ theme. We must move beyond the ‘professionals know best’ narrative. Delahooke (2019) has argued that professionals need to broaden their frameworks to move ‘beyond behaviours’ when supporting individuals (Bowring et al., 2019; van den Akker et al., 2021). This study would suggest that there needs to be a change in how we think about behaviors of concern.

This study employed qualitative methodologies to help understand the lived experiences of parents. Focusing on a relatively small sample size enabled the researchers to gather in-depth and meaningful data relating to participants’ views on Low Arousal training and the impact of applying this approach on family life. Semi-structured interviews allowed parents to bring topics pertinent to their experiences that we had not specifically asked about. This fitted well with our ‘bottom-up’ approach to data analysis and enabled the emergence of themes not anticipated by the researchers. In this case, practice-based evidence focused on parents’ voices and enabled the identification and implementation of solutions that can be applied in real-world settings (Ammerman et al., 2014).

## Limitations and implications

Several limitations of this study must be acknowledged. First, the authors recognize that the working relationship between interviewed families and the researchers may have influenced the participants to report more positive experiences of utilizing the Low Arousal Approach. Second, families who have reportedly found the application of their training effective and helpful may have done so due to their preexisting, ‘non-confrontational’ parenting style. It is possible that the families in this qualitative study may have a positively skewed view of the Low Arousal Approach. It would appear that studies focusing on a greater and more diverse sample would be needed to examine the generalizability of the results. Third, the authors recognize objectivity issues arising from their expertise in this area. While we strived to remain critically reflective and impartial in our analyses, we acknowledge that our prior understanding of the issues families often encounter may have influenced our interpretation.

There are a number of implications that warrant further investigation. Qualitative methodologies do provide a perspective on processes (Crane et al., 2021). There are a number of implications for quantitative approaches in the future. This study has not evaluated the specific elements of training in Low Arousal Approaches. A series of training studies based on randomized samples would be a sensible next step. A future study may need to focus more on detail about what specific elements of training are most useful for families. The relationship between family stress and managing day-to-day situations will require further exploration. Developing consistency would appear to require professionals to adjust their practice (Argumedes et al., 2018). This study would suggest that there are some interpersonal conflicts that relate to the application of the Low Arousal Approach. A possible mechanism to reduce this conflict should involve a stronger emphasis on training between professionals and family members in day-to-day crisis management. Finally, the construct of Low Arousal Approaches (Elvén, 2010; McDonnell, 2010, 2019) needs to be placed in context with other interventions, such as Positive Behavior Supports (PBS).

## Conclusion

Family training in Low Arousal Approaches is considered to be necessary, but not sufficient, to achieve behavior change in its own right (McDonnell, 2010). From the perspective of parents, families who receive training in these approaches have a greater sense of empowerment and confidence in managing behaviors of concern. Encouraging a focus on reducing restrictions and avoiding punitive consequences appears to be empowering. Changing the balance of power from professionals to consumers would appear to be the next step. Parents trained in these approaches suggest that reducing stress in families that must manage behaviors of concern needs to have a multifaceted approach. Professionals and supporters of families need to be able to equip people with skills to manage these situations (Doubet and Ostrosky, 2015). From an empirical perspective, Low Arousal Approaches are still in their infancy. However, based on this limited evidence, it appears that training in crisis management and employing Low Arousal Approaches to manage distressed behavior has some utility.

## Data availability statement

The original contributions presented in the study are included in the article/Supplementary material, further inquiries can be directed to the corresponding author.

## Ethics statement

The studies involving humans were approved by Birmingham City University Ethics Committee. The studies were conducted in accordance with the local legislation and institutional requirements. The participants provided their written informed consent to participate in this study.

## Author contributions

AM: Writing – original draft, Supervision, Methodology, Formal analysis. AP: Writing – original draft, Methodology, Conceptualization. SB-P: Writing – original draft, Methodology, Data curation. KM: Writing – original draft, Data curation. TK-J: Writing – review & editing, Methodology, Data curation. MM: Writing – review & editing.
