# Supplementary material for: Families’ experiences of the Low Arousal Approach: a qualitative study
Source: Front Psychol. 2024 Mar 21;15:1328825. doi: 10.3389/fpsyg.2024.1328825 (PMC11002904; doi:10.3389/fpsyg.2024.1328825)
Supplement: Supplementary file 1 [file Data_Sheet_1.pdf]

Appendices

Appendix A- Research Consent Form

Consent Form

1. By signing this consent form, I agree to take part in this research study.

2. I have read and understood the Participant Information Sheet that was given to me alongside this consent form.

3. The reason for and any possible effects of this study have been explained to me, and I understand that the study involves the following;

    - Participants being asked to complete an interview with a researcher regarding their experiences of applying the Low Arousal Approach

4. I understand that participating in this research study involves the risk of not gaining any new knowledge or confidence.

5. I understand that all the research data will be securely stored on a password-protected database for one year from the publication of the study results and will then be destroyed.

6. Any questions that I have asked have been answered to my satisfaction

7. I understand that the researchers will maintain confidentiality and that any information I supply to the researchers will be used **only** for the purposes of this research.

8. I understand that the results of the study will be published, so that I cannot be identified as a participant.

9. I understand that my participation is voluntary, and I may withdraw at any point without any effect.

10. I understand that I will not be able to withdraw my data after completing the study as it will be stored anonymously.

Participant's full name:

____

Participant's signature:

____

Date: ______________________________________

Statement by the investigator

I have explained the research study and the implications of participation in it to this volunteer and I believe that the consent gained is informed and that he/she understands the implications of their participation

If the investigator has not had an opportunity to talk to participants prior to them participating, the following must be ticked;

The participant has received the Participant Information Sheet where my details have been provided so participants have had the opportunity to contact me prior to consenting to participate in the research study.

Investigator's full name:

____

Investigator's signature:

____

Date: ______________________________________

Appendix B- Participant Information Sheet

Participant Information Sheet

1. **Invitation**
   You are invited to participate in a research study examining parents' experiences of applying the Low Arousal Approach.

   This study is being conducted by:

   - Assistant Psychologists and Research Officer, Studio 3
   - Andrea Page, Associate Professor, Birmingham City University, West-Midlands

2. **What is the purpose of this study?**
   The aim of this study is to explore parents' experiences of applying the Low Arousal Approach to behaviours of concern displayed by their child after having training in the approach by Studio 3.

3. **Why have I been chosen to participate?**
   You are eligible to take part in this research study because you have received training in the Low Arousal Approach by Studio 3 and have experience applying this approach to behaviours of concern.

4. **What will I be asked to do?**

   - Consent to take part in this research study will be sought
   - Participants will be contacted by the researchers to take part in a telephone interview discussing their experiences of applying the Low Arousal Approach
   - No access to personal records will be required, and all questionnaires will be coded to maintain the confidentiality of the participant
   - There will be no requirement to film, but telephone calls will be recorded as data

5. **Are there any possible benefits from participation in this study?**
   We cannot guarantee or promise that you will receive any benefits from this research however possible benefits may include an increased understanding of how to use the Low Arousal Approach when faced with behaviours of concern.

6. **Are there any possible risks from participation in this study?**
   There is a risk that you do not gain any further knowledge from participation in this research study. You may feel that some of the questions we ask are stressful or upsetting. If you do not wish to answer a question, you may skip it and go onto the next question or stop immediately.

All data for this study is collected by interviews via the telephone. There are no group discussions or questionnaires involved in this research study.

7. **What if I change my mind during or after the study?**
   If you do consent to participate, you may withdraw at any point. If you decide to withdraw from the project, please notify a member of the research team before you withdraw. Data already collected will remain in the study, but no further data will be collected.

8. **What will happen to the information when this research study is over?**
   All information will be reviewed by Assistant Psychologists at Studio 3, information and data from the telephone interviews will be stored as files on a data stick, encrypted, password protected and secured in a locked cabinet. No personal data is required, Studio 3 will share information with Andrea Page from Birmingham City University via secure email. Data will be stored for three years in total.

9. **How will the results be published?**
   It is anticipated that the results of this research study will be published and/or presented in a variety of forums. The individuals and stakeholders engaging in the study will be emailed a copy of the final report and a brief presentation of the research and its findings. Participants can expect a copy of the final results and summary within a year of completing their telephone interview with the researchers.

10. **Who should I contact if I have questions about this research study?**
    Stephanie Bews-Pugh can be contacted on stephanieb@studio3.org

Thank you for taking the time to read this.
If you are interested in taking part, please read and sign the Consent Form and return it to the researchers.

Appendix C- Interview Schedule

Semi-structured interview questions

Background questions

1. What training courses in the low arousal approach have you attended in the last 2 years?
2. How long was this (each) course?
3. Where did the training take place?
4. Who provided the training (i.e. organisation name)?

Questions about the contents of the training

5. What was the most valuable thing you learnt from the training course (each course) and why?
6. What was the least valuable thing you learnt from the training course (each course) and why?

Questions about application of the low arousal approach

7. Describe a situation/situations you managed successfully using the low arousal approach
8. How easy was it to apply the low arousal approach in these situations?
9. Describe a situation/situations you did not manage as well using the low arousal approach
10. What was difficult about applying the low arousal approach in these situations?

Questions about the perceived impact of the training

11. Have you improved on your parenting skills following training in the low arousal approach?
    Probe: In what ways?
12. Has the low arousal training improved your relationship with your child who presents with behaviours of concern?
    Probe: In what ways?
13. Has the low arousal training improved your relationship with your partner?
    Probe: In what ways?
14. Has training in the low arousal approach increased your awareness in understanding the impact of your behaviour on your child?
    Probe: In what ways?
15. Has training in the low arousal approach increased your confidence in managing your child’s behaviours of concern?
    Probe: In what ways?
16. Have you any other comments that you would like to add?
